# Complete mitochondrial genome of the hybrid grouper *Hyporthodus septemfasciatus* (♀)×*Epinephelus moara* (♂) (Perciformes, Serranidae) and results of a phylogenetic analysis

**DOI:** 10.1080/23802359.2021.1881840

**Published:** 2021-03-11

**Authors:** Yong Hwi Kim, Jong Yeon Park, Duc Tam Huynh, Kang Rae Kim, In-Chul Bang

**Affiliations:** a Department of Life Science and Biotechnology, Soonchunhyang University, Asan, Republic of Korea; b Aqua Biotech Co., Ltd., Daejeon, Republic of Korea

**Keywords:** Serranidae, *Hyporthodus septemfasciatus*, *Epinephelus moara*, hybrid, mitogenome

## Abstract

The complete mitochondrial genome of the hybrid grouper *Hyporthodus septemfasciatus* (♀)×*Epinephelus moara* (♂) was obtained by next-generation sequencing. The mitochondrial genome was 16,499 bp long, consisting of 13 protein-coding genes, 22 transfer RNA genes, two ribosomal RNA genes, and a control region (D-loop). The overall base composition is 28.62% A, 28.27% C, 16.27% G, and 26.84% T with 55.46% A + T. In the maximum-likelihood (ML) phylogenetic analysis, the hybrid grouper belonged to the same clade as *H*. *septemfasciatus* (maternal inheritance).

The convict grouper, *Hyporthodus septemfasciatus* Thunberg, 1793 and kelp grouper, *Epinephelus moara* Temminck & Schlegel, 1842 belong to the subfamily Epinephelinae, family Serranidae, order Perciformes. Both are commercial fish species popular for artificial propagation and aquaculture, mainly in China, the Republic of Korea, and Japan (Sabate et al. 2009; Tian et al. 2013; Noh and Yoon 2019). The groupers produce hybrids, which are used in the aquaculture industry because of advantages such as fast growth rates, tolerance of disease, and a wide range of water temperatures (Kiriyakit et al. 2011).

In this study, hybrid groupers *H*. *septemfasciatus* (♀)×*E*. *moara* (♂) were produced through artificial insemination at the Soonchunhyang University Marine Fisheries Research Institute (36°61′959″N 126°33′552″E) in July 2020. Samples were collected immediately after hatching and preserved in 99.9% ethyl alcohol. This hybrid has high nutritional and economic value (Li et al. 2016).

Since hybrid groupers are difficult to distinguish morphologically, the complete mitochondrial genome of *H*. *septemfasciatus* (♀)×*E*. *moara* (♂) reported here provides useful information for molecular phylogenetic and taxonomic studies.

Genomic DNA (gDNA) was extracted from entire larva using a HiGene™ Genomic DNA Prep Kit (BioFact, Daejeon, Republic of Korea), and extracted gDNA was stored at the specimen storage facility of Soonchunhyang University (voucher no. SUC25880).

A qualified library was constructed by sequencing 2 × 150 bp paired-end reads on an MGISEQ-2000 platform (MGI Tech, Shenzhen, China) using extracted gDNA. In addition, all raw reads were deposited in the GenBank Sequence Read Archive (SRA; SRR13279997). The obtained mitochondrial genome sequences were assembled using Geneious R11 (Kearse et al. 2012).

The complete mitochondrial genome of the hybrid grouper *H*. *septemfasciatus* (♀)×*E*. *moara* (♂) (GenBank accession no. MW151226) is 16,499 bp long and includes 13 protein-coding genes (PCGs), 22 transfer RNA (tRNA) genes, two ribosomal RNA (rRNA) genes, and a control region (D-loop). The overall base composition is 28.62% A, 28.27% C, 16.27% G, and 26.84% T with 55.46% A + T, which is similar to the base content and AT bias of other vertebrate mitochondrial genomes (Saccone et al. 1999).

The 12S rRNA (956 bp) is located between *tRNA^Phe^* and *tRNA^Val^*, and the 16S rRNA gene (1709 bp) between *tRNA^Val^* and *tRNA^Leu^*. Of the 13 PCGs, 11 have ATG start codons; those of the *co1* and *atp6* genes are GTG and CTG, respectively. The latter is infrequently the start codon of the *atp6* and *nd2* genes in vertebrate and ascidian mitochondrial genomes (Donath et al. 2019). Three of these PCGs terminate with incomplete stop codons (*co2*, *nd4*, and *cytb*), while the remaining 10 end with complete stop codons (TAA or TAG). The control region (792 bp) is located between *tRNA^Pro^* and *tRNA^Phe^*.

All mitochondrial genome sequences used in the phylogenetic analysis were downloaded from the National Center for Biotechnology Information, aligned using MAFFT ver. 7.450 (Katoh et al. 2002; Katoh and Standley 2013), and analyzed.

A phylogenetic tree was constructed based on 13 PCGs from the complete mitochondrial genome of major species in the genera *Hyporthodus* and *Epinephelus*. GTR + I+G was confirmed to be the best-fitting evolutionary model based on the corrected Akaike information criterion (AICc), obtained using jModelTest 2.1.10 (Guindon and Gascuel 2003; Darriba et al. 2012). The GTR + I+G model was used for maximum-likelihood (ML) estimation, based on an analysis conducted in RAxML 8.0.11 (Stamatakis 2014) with 1000 bootstrap replicates. In addition, Bayesian inference (BI) tree was run for 1,000,000 generations using MrBayes 3.2.7 (Ronquist et al. 2012). Three species of *Cephalopholis* and *Variola* in the subfamily Epinephelinae were used as outgroups (Figure 1).

**Figure 1.**
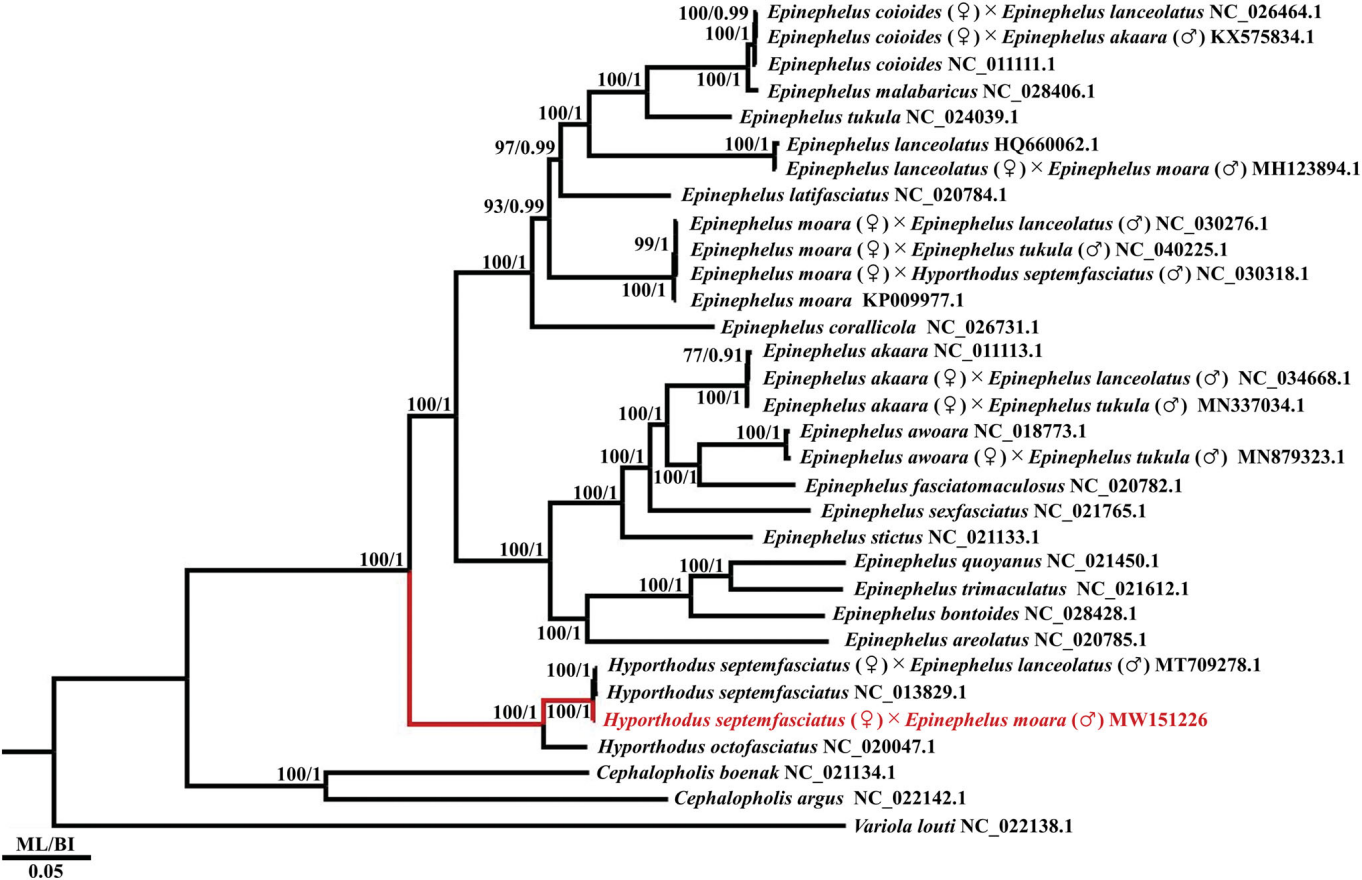
The phylogenetic tree of the genus *Hyporthodus*, obtained from maximum likelihood (ML) and Bayesian inference (BI) analyses of 13 protein-coding genes (PCGs). Bootstrap values above 70% in the ML analysis and posterior probabilities above 0.90 in the BI analysis are shown at the base of each node. The best-fitting evolutionary model was the GTR + I+G model. The GenBank accession numbers follow the scientific names.

In the phylogenetic tree of the subfamily Epinephelinae, the genera *Epinephelus*, *Hyporthodus*, *Cephalopholis*, and *Variola* each formed a clade, supporting the current taxonomy. The mitochondrial genome of the hybrid grouper *H*. *septemfasciatus* (♀)×*E*. *moara* (♂) obtained here was in the same clade as that of *H*. *septemfasciatus* (maternal inheritance), in accordance with maternal inheritance of mitochondrial DNA in eukaryotes, similar to other hybrid groupers (Sato and Sato 2012). This mitochondrial genome will improve the database for the subfamily Epinephelinae, and sheds light on the molecular phylogeny and taxonomy.

## Data Availability

The data that support the findings of this study are openly available in GenBank of NCBI at https://www.ncbi.nlm.nih.gov, reference number MW151226. The associated BioProject, SRA and Bio-Sample numbers are PRJNA686855, SRR13279997, and SAMN17126625, respectively.
